# Voluntary disclosure, banking stability, and AI-augmented forensic accounting: an exploratory econometric and machine-learning study of Palestinian banks

**DOI:** 10.3389/fdata.2026.1914133

**Published:** 2026-09-14

**Authors:** Bahaa Subhi Razia, Najwan Ibrahim Jadallah, Qasim Zureigat, Reem Khamis, Bahaa Subhi Awwad

**Affiliations:** 1 Department of Industrial and Logistics Management, Palestine Technical University – Kadoorie, Tulkarm, Palestine; 2 Department of Computerized Banking and Financial Science, Faculty of Business and Economics, Palestine Technical University – Kadoorie, Tulkarm, Palestine; 3 Sulaiman AlRajhi School of Business, Sulaiman AlRajhi University, Al Bukayriyah, Saudi Arabia; 4 Department of Business Administration, University College of Bahrain, Manama, Bahrain; 5 Department of Accounting, Finance and Banking, College of Business and Finance, Ahlia University, Manama, Bahrain

**Keywords:** anomaly detection, artificial intelligence, banking stability, forensic accounting, instability-risk screening, machine learning, voluntary disclosure, *Z*-score

## Abstract

**Introduction:**

Information asymmetry between bank managers and external stakeholders is a common relationship between financial-statement fraud and banking instability.

**Methods:**

This study combines an AI-augmented forensic accounting framework with voluntary disclosure, banking stability indicators, and exploratory machine learning (ML) approaches. The study integrates fixed-effects regression with Logistic Regression, Random Forest, XGBoost, Isolation Forest, and SHAP-based explainability using panel data from the whole population of seven banks listed on the Palestine Exchange (2019–2025).

**Results:**

According to the econometric results, there is a conditional rather than a uniform relationship between voluntary disclosure and financial stability, with variation by bank size, age, and leverage. Additionally, the exploratory machine-learning analyses indicate that nonlinear approaches could help find unusual bank-year records and instability-risk patterns that are not fully captured by traditional linear models. SHAP analysis enhanced the interpretability of model classifications, and ensemble approaches outperformed Logistic Regression in cross-validation within this small sample. The machine-learning results are considered as exploratory proof-of-concept evidence rather than externally confirmed predictive outcomes due to the small sample size and lack of independently verified fraud labels.

**Discussion:**

Overall, the study shows how AI-augmented forensic accounting can enhance supervisory prioritization, instability-risk screening, and the expert assessment of anomalous observations in institutionally unstable banking contexts, thereby complementing traditional econometric analysis.

## 1 Introduction

Financial statement fraud and banking instability are viewed as distinct problems by several research communities. While forensic accountants and fraud detectives investigate whether a company's reported stats misrepresent its underlying condition, banking-stability scholars wonder whether a bank's true state is solid enough to withstand shocks. All avenues of investigation, however, begin with the same structural condition: bank managers have greater knowledge about asset quality, credit risk, and the sustainability of reported earnings than do depositors, investors, or even supervisors. Both inadvertent opacity and intentional concealment are made possible by this information asymmetry (Akerlof, 1970; Stiglitz and Weiss, 1981; Healy and Palepu, 2001; Christensen et al., 2021). From the perspective of an external stakeholder, a bank that conceals declining asset quality to avoid a supervisory downgrade and a bank that merely discloses insufficient information to be properly assessed by outsiders appear strikingly similar: both leave the stakeholder unable to discern between a truly stable institution and a fragile one until it is too late.

The main point of this work is motivated by this observation. The set of accounting and non-accounting information that bank management decides to disclose beyond regulatory minimums is known as voluntary disclosure. It has long been researched as a factor that determines information asymmetry and, consequently, the cost of capital, investor confidence, and market discipline (Healy and Palepu, 2001; Christensen et al., 2021). Supervisors and scholars have long applied banking-stability measures such as the Bank *Z*-score and the CAMELS framework to evaluate the soundness and resilience of financial institutions. Although conceptually related to the broader score-based tradition initiated by (Altman 1968), the Bank *Z*-score used in banking research is mathematically distinct from Altman's original corporate-failure model (Abdul Rahman and Masngut, 2014; Lepetit and Strobel, 2013, 2015; Awwad and Razia, 2021). Several models are considered popular in this regard, including the Beneish *M*-score (Beneish, 1999), the Dechow *F*-score (Dechow et al., 2011), and machine-learning-based anomaly screening models that have demonstrated superior performance in identifying financial statement irregularities (Perols, 2011). It is argued that recent developments in artificial intelligence (AI) and machine learning (ML) offer a natural way to integrate these three streams of work disclosure research, banking-stability research, and fraud-detection research into a single, more potent surveillance framework, especially in institutionally fragile banking markets where supervisory resources are limited and disclosure practices are uneven.

A true empirical analysis of the Palestine Exchange's (PEX) banking industry, which is summed up in Section 3, was used to develop this claim. An 11-item voluntary disclosure index (VDI) covering topics like financial inclusion, cybersecurity and customer-data-protection policy, gender diversity, green financing, and senior-management continuity is constructed using panel data on the seven PEX-listed commercial and Islamic banks from 2019 to 2025 (49 bank-year observations). The underlying analysis then links this index to banking stability as determined by the *Z*-score and the six CAMELS framework dimensions (capital adequacy, asset quality, management efficiency, earnings, liquidity, and market-risk sensitivity). From a straightforward linear-effects perspective, the results appear perplexing: voluntary disclosure is positively and significantly associated with management efficiency and earnings, negatively (and insignificantly) associated with the *Z*-score, and its association with capital adequacy and asset quality is not constant but depends on bank size, age, and leverage through statistically significant interaction effects. Despite careful specification, traditional fixed-effects regression is structurally limited in its capacity to fully characterize this conditional and interaction-dependent pattern, which is precisely the kind of structure that artificial intelligence techniques—such as ensemble tree-based learners, anomaly-detection algorithms, and text-mining techniques applied to disclosure narratives—are well suited to capture.

This is how the rest of the paper goes. The theoretical underpinnings of voluntary disclosure, banking stability, anomaly screening, and machine learning are developed and critically reviewed in Section 2. The econometric data from Palestinian banks is shown in Section 3. The classification, feature-importance, SHAP, and anomaly-detection findings are reported in Section 4 along with a description of the exploratory machine-learning process. In Section 5, it is explained why traditional linear econometric models might not adequately reflect nonlinear forensic-risk patterns, although being crucial for statistical inference. An AI-augmented forensic accounting paradigm with empirical support is developed in Section 6. Future large-sample validation ideas are presented in Section 7. The consequences for banking supervision, regulatory capacity-building, and forensic accounting practice are covered in Section 8. The study's shortcomings and a plan for more empirical validation are presented in Section 9, and the paper is concluded in Section 10. To determine whether nonlinear disclosure–stability patterns can be found in the Palestinian banking industry, this study uses exploratory machine-learning approaches as a supplementary analytical layer to traditional econometric analysis.

### 1.1 Research questions

This study addresses the following research questions:

RQ1: Does voluntary disclosure influence banking stability in Palestinian banks?

RQ2: Is the relationship between voluntary disclosure and banking stability conditional upon bank-specific characteristics such as size, age, and leverage?

RQ3: Can machine-learning techniques complement traditional econometric methods in identifying instability-risk patterns and forensic accounting signals?

RQ4: How can AI-augmented forensic accounting support banking surveillance in institutionally fragile markets?

### 1.2 Research objectives

The primary objectives of this study are:

To examine the relationship between voluntary disclosure and banking stability.

To investigate the moderating role of bank characteristics.

To explore the usefulness of machine-learning techniques for identifying instability-risk patterns. To develop an AI-augmented forensic accounting framework applicable to fragile banking environments.

### 1.3 Theoretical and practical contributions

The study contributes theoretically by integrating Information Asymmetry Theory, Signaling Theory, and forensic accounting literature into a unified disclosure–stability–surveillance framework. Practically, it provides regulators, auditors, and forensic accountants with an AI-augmented approach for enhancing early-warning systems and supervisory monitoring in small and emerging banking markets.

The article is positioned as an exploratory empirical study with an empirically informed framework contribution rather than as either a purely conceptual article or a fully validated predictive study. The panel-data and machine-learning analyses were implemented using the final dataset of 49 bank-year observations and therefore constitute empirical analyses. However, because the dataset is small and no independent fraud or supervisory-enforcement labels are available, the reported machine-learning results are not interpreted as externally validated evidence of fraud-detection performance. Instead, they provide preliminary, context-specific evidence regarding the feasibility of integrating voluntary disclosure, banking-stability indicators, anomaly screening, and explainable machine learning within a unified forensic-accounting framework. The econometric and machine-learning findings therefore motivate and inform the framework, while the framework's broader predictive and operational validity remains a matter for future large-sample, out-of-time, and cross-country validation.

Figure 1 shows the research design and the analytical framework. This study starts with identifying the research problem, which includes information asymmetry, non-disclosure and instability risk in Palestinian banks. It investigates its relationship with banking stability using traditional econometric analysis and then expands the analysis using AI-based procedures, explainable AI, and anomaly-detection techniques. This plays an important role in supporting AI-augmented forensic accounting, identifying potential risks, and enhancing stability and oversight through regulatory decision-making.

**Figure 1:**
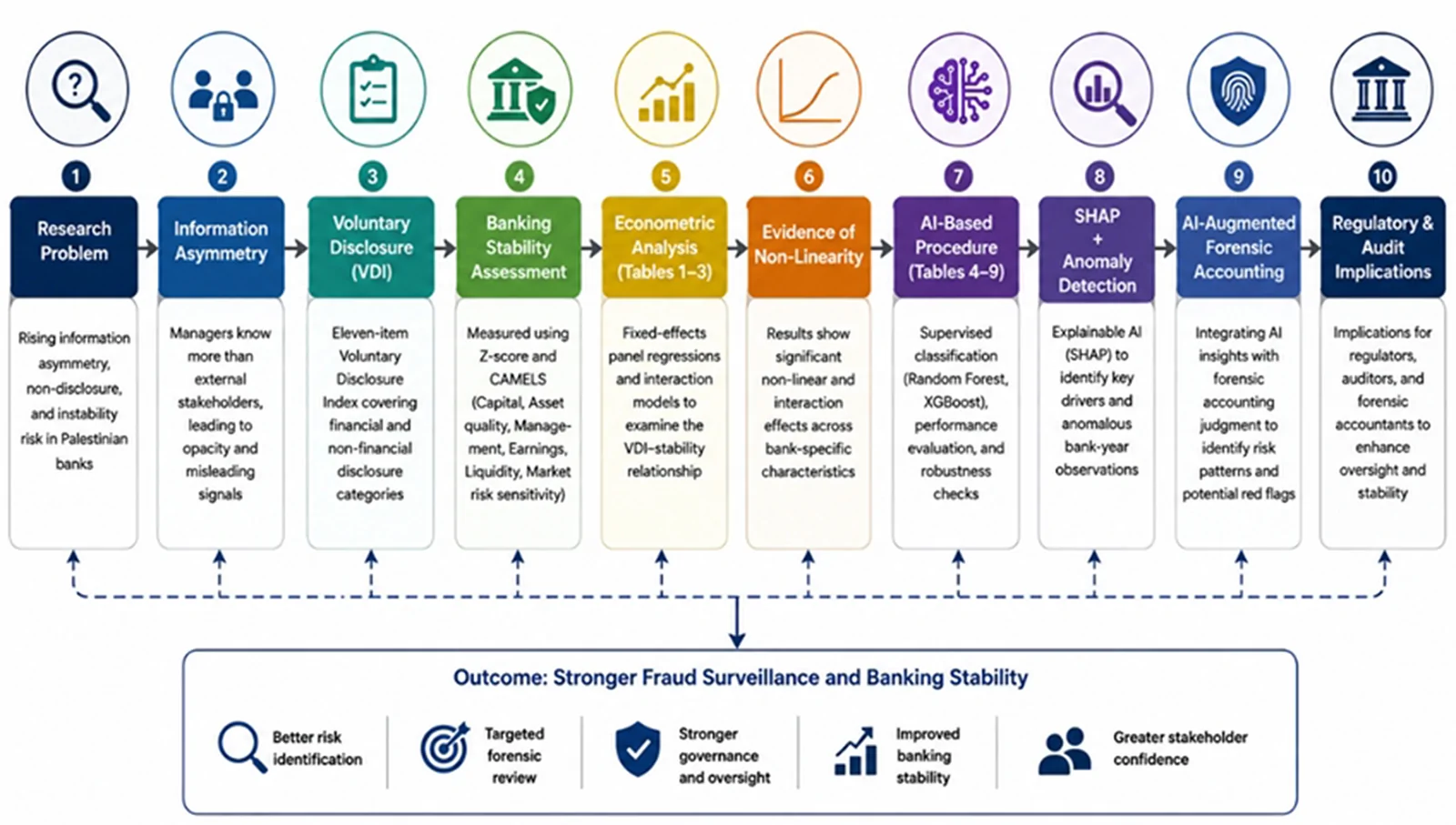
Research design and analytical framework. *Source* Developed by the authors. Conceptual illustration developed for explanatory purposes.

## 2 Background: disclosure, stability, and forensic-risk screening as an integrated analytical problem

### 2.1 The disclosure–concealment–instability triangle

Information Asymmetry Theory, Signaling Theory, and the forensic-accounting viewpoint are all integrated into the study's theoretical framework. According to information asymmetry theory, external stakeholders are unable to effectively assess a bank's underlying financial position due to disparities in information availability. Depositors, investors, auditors, and regulators may be unable to differentiate financially sound institutions from vulnerable ones when bank managers have superior knowledge of asset quality, credit exposure, earnings sustainability, and operational risks (Akerlof, 1970; Stiglitz and Weiss, 1981). According to Signaling Theory (Spence, 1973; Ross, 1977; Bhattacharya, 1979), financially sound organizations have an incentive to provide reliable information that sets them apart from weaker institutions. Therefore, voluntary disclosure can serve as an indicator of institutional soundness, openness, and good governance in the banking industry. According to disclosure studies, increased openness can lessen information asymmetry, improve market discipline, reduce uncertainty, and promote financial stability (Healy and Palepu, 2001; Christensen et al., 2021). However, just because more information is made public does not mean that disclosure is a reliable indication. The disclosed content's relevance, specificity, verifiability, and consistency determine its informational value.

However, there may be more to the relationship between transparency and financial stability than just a straightforward positive and monotonic correlation. The same incentives that promote credible signaling may also promote selective, symbolic, or strategically inadequate reporting; voluntary disclosure is a managerial decision. While providing little information about credit concentration, loan quality, liquidity pressure, or other financially diagnostic risks, a bank may reveal positive and comparatively inexpensive information, such as training initiatives, community programs, digital-service statistics, or general sustainability commitments. From the standpoint of forensic accounting, this leads to a disclosure-concealment issue: information that is crucial for assessing financial soundness may be omitted or concealed alongside what appears to be substantial reporting. The categories of earnings manipulation, material misstatement, financial-statement fraud, and accounting anomalies are used in related research to analyze similar reporting practices (Beneish, 1999; Dechow et al., 2011; Perols, 2011; Schreyer et al., 2019). Therefore, a bank may appear transparent without necessarily lowering information asymmetry if it publishes a lot of non-financial disclosures but gives little information on declining financial fundamentals. Therefore, one of the main responsibilities of forensic accounting and banking oversight is to distinguish genuine disclosure from selective or perhaps deceptive disclosure. The main constructs employed in this study should be differentiated for conceptual accuracy.

When bank insiders and external stakeholders have different access to pertinent information, this is known as information asymmetry. A disclosure gap does not prove deliberate concealment or misrepresentation on its own; rather, it describes the lack, incompleteness, or insufficient specificity of information needed for external evaluation. Financial vulnerability as determined by the *Z*-score and CAMELS proxies created by researchers is referred to as banking instability. An observation that significantly deviates from the multivariate patterns found in the dataset is known as a statistical anomaly, and it should be seen as a screening signal rather than proof of wrongdoing. While verified financial-statement fraud necessitates independent documentary, supervisory, judicial, or enforcement evidence, misstatement risk refers to situations that can warrant additional professional investigation. As a result, the current methodology does not establish if fraud has taken place; rather, it finds instability-risk patterns and unusual findings that can call for forensic examination.

### 2.2 A shared analytical lineage: from the *Z*-score to the *M*-score to machine learning

Banking-stability assessment and financial-statement-anomaly screening have developed as distinct research streams, although both use combinations of financial and non-financial indicators to distinguish sound observations from potentially problematic ones. The Bank *Z*-score used in banking research combines profitability, capitalization, and earnings volatility to measure a bank's distance from insolvency. It is conceptually and mathematically distinct from Altman's (1968) coefficient-weighted corporate-failure *Z*-score. The CAMELS framework provides a complementary assessment of bank soundness through capital adequacy, asset quality, management efficiency, earnings, liquidity, and sensitivity to market risk. Alternative Bank Z-score constructions and volatility windows may materially affect measured insolvency risk and should therefore be specified transparently (Lepetit and Strobel, 2013, 2015). More recent studies have applied bank-specific indicators and machine-learning methods to improve the identification of banking crises and insolvency risk (Beutel et al., 2019; Petropoulos et al., 2020).

Research on financial statement fraud uses a similar analytical framework. While the Dechow *F*-score calculates the probability of significant financial misstatements, the Beneish *M*-score employs accounting indications to spot possible earnings manipulation (Beneish, 1999; Dechow et al., 2011). These methods show that financial ratio combinations can contain signals that are not immediately apparent from individual accounting measurements. However, complex nonlinearities, threshold effects, and higher-order interactions may be harder for traditional score-based and logistic models to capture since they typically impose fixed functional forms.

By enabling the identification of correlations between financial ratios, disclosure indicators, and bank characteristics without the need to predetermine every interaction or threshold, machine learning expands this analytical lineage. The increasing application of data mining and machine-learning approaches for financial fraud and anomaly screening has been reported in earlier reviews (Ngai et al., 2011; West and Bhattacharya, 2016). The ability of statistical and machine-learning algorithms to identify financial-statement fraud varies significantly, as demonstrated by (Perols 2011). Later, (Bao et al. 2020) showed that machine-learning techniques can detect accounting errors more accurately than traditional fraud-prediction models. (Beutel et al. 2019) and (Petropoulos et al. 2020) show that by capturing nonlinear and interaction-dependent risk structures, machine-learning algorithms can enhance the prediction of banking crises and insolvency in banking research. These results lend credence to the idea that ensemble techniques like Random Forest and XGBoost should be used in conjunction with traditional econometric analysis rather than in place of statistical inference.

When trustworthy fraud labels are not provided, unsupervised anomaly-detection techniques offer an extra forensic benefit. To find observations that significantly differ from common patterns, autoencoder-based and related anomaly-detection approaches have been used to accounting and bookkeeping data (Schreyer et al., 2019; Mashiko et al., 2025). These techniques are especially useful in banking settings when verified fraud cases, enforcement actions, or supervisory downgrades are rare, private, or unavailable for empirical examination. Detected abnormalities can serve as risk signals that direct auditors, forensic accountants, and supervisors toward findings that need further examination rather than proving wrongdoing. Beyond traditional financial ratios, machine learning can also increase the amount of data available for forensic examination.

Conventional disclosure indices frequently simplify reporting procedures to binary or aggregate scores, possibly ignoring linguistic concealment, tone, specificity, uncertainty, and repetition. According to a recent auditing study, financial narratives can yield additional information beyond what financial statistics alone can provide when machine learning and textual analysis are combined (Bochkay et al., 2023). This capacity is crucial because, even when traditional accounting indicators stay within permitted bounds, selective disclosure and linguistic ambiguity may indicate reporting risk.

Nevertheless, the existing literature remains fragmented. Disclosure studies primarily examine information asymmetry, market discipline, and reporting consequences but rarely incorporate disclosure patterns into forensic-risk detection. Banking-stability studies rely on CAMELS indicators, *Z*-scores, and other financial measures but generally give limited attention to the strategic or selective nature of voluntary disclosure. Fraud-detection and machine-learning studies can identify nonlinearities and anomalous observations, yet they rarely integrate voluntary disclosure quality with bank-specific stability measures in small and institutionally fragile markets. Consequently, each literature provides only a partial explanation of the disclosure–stability–forensic-risk relationship.

This study addresses that gap by integrating voluntary disclosure, *Z*-score and CAMELS-based banking-stability measures, conventional panel-data econometrics, supervised machine learning, unsupervised anomaly detection, and explainable AI within a unified disclosure–stability–surveillance framework. This integration allows the study to examine average econometric relationships while also identifying non-linear risk patterns, influential interactions, and anomalous bank-year observations that may warrant additional forensic or supervisory examination. To the best of our knowledge, this combined approach has not previously been applied to the Palestinian banking sector or to a comparable fragile-market banking setting.

## 3 Empirical motivation: evidence from Palestinian Banks (2019–2025)

This section presents the preliminary econometric evidence derived from the complete population of seven banks listed on the Palestine Exchange during 2019–2025. The analysis provides context-specific empirical evidence regarding the disclosure–stability relationship and informs the subsequent machine-learning analysis and AI-augmented forensic-accounting framework; it is not presented as broadly generalizable or causal validation.

The study covers the complete population of banks listed on the Palestine Exchange throughout the 2019–2025 study period rather than a selected subsample. Therefore, the limited number of cross-sectional units reflects the actual size and institutional structure of the Palestinian listed banking sector. Nevertheless, the resulting panel of 49 bank-year observations constrains statistical power, limits the number of parameters that can be estimated simultaneously, and requires particular caution when interpreting interaction effects. Instead of entering the interaction factors concurrently into a single saturated model, they were evaluated in distinct model specifications to minimize over-parameterization. Therefore, rather than being estimates that may be universally applied to bigger or institutionally distinct banking markets, the findings should be viewed as context-specific data on the listed Palestinian banking sector. Therefore, the machine-learning results are seen as exploratory evidence that must be validated using bigger pooled banking datasets, while the econometric results are largely understood in terms of the direction, consistency, and conditional nature of the observed connections.

A manually created binary disclosure-presence index based on the seven banks published annual reports was used to gauge voluntary disclosure. The unit of study was the bank-year annual report, and all 49 annual reports from 2019 to 2025 were looked at. Eleven disclosure categories—financial inclusion, SME financing, corporate social responsibility, customer satisfaction, forward-looking plans, digital platform usage, cybersecurity policy, customer data protection, gender diversity in management, green financing policy, human capital development, and management continuity—were included in the final coding schedule. The last category contributed a maximum score of one to each bank-year observation since it was handled as a single composite category rather than two distinct indicators.

Only when the annual report had a clear and recognizable disclosure that met the predetermined operational condition was each category marked 1, and when no qualifying disclosure was discovered, it was coded 0. The annual report and its distinct sections on finance, governance, risk management, sustainability, corporate responsibility, and human capital were all coded. Statements that were purely promotional, general, aspirational, or boilerplate were not coded unless they mentioned a particular program, practice, policy, quantifiable activity, institutional arrangement, or reported outcome that was pertinent to the category. Repeated phrasing across years was not taken as proof of better disclosure quality; rather, it was solely marked as disclosure presence for the relevant bank-year. The operational criteria are given in Table 1, and the main annual-report locations examined for each category are included in Appendix Table A2 along with qualifying instances and excluded boilerplate samples.

**Table 1:** Definitive coding schedule for the 11-item voluntary disclosure index.

No.	Disclosure item	Coding criterion for a score of 1
1	Financial inclusion	Explicit disclosure of initiatives, policies, services, or programmes aimed at expanding access to financial services
2	SME financing	Explicit disclosure of financing, credit facilities, programmes, or support directed toward small and medium-sized enterprises
3	Corporate social responsibility	Disclosure of identifiable social, community, charitable, or broader corporate-responsibility activities
4	Customer satisfaction	Disclosure of customer-satisfaction measurement, surveys, complaint-management systems, or related performance information
5	Forward-looking plans	Disclosure of identifiable future strategies, objectives, expansion plans, or planned institutional developments
6	Digital-platform usage	Disclosure of digital-banking platforms, electronic services, mobile banking, or measurable digital-service usage
7	Cybersecurity policy	Explicit disclosure of cybersecurity policies, controls, systems, risks, or related institutional measures
8	Customer-data protection	Explicit disclosure of privacy, confidentiality, or customer-data-protection policies and procedures
9	Gender diversity in management	Disclosure of female representation, gender diversity, or gender-related policies at management or governance levels
10	Green-financing policy	Disclosure of green financing, environmental lending, sustainable-finance products, or related environmental policies
11	Human-Capital Development and Management Continuity	Explicit disclosure of formal employee-training or professional-development programmes and/or information concerning the continuity, succession, or stability of senior management. The two related elements constitute one composite category and generate a maximum score of one per bank-year

Each category was coded 1 when an explicit and identifiable disclosure satisfying the stated operational criterion appeared in the bank's annual report and 0 otherwise. Generic, promotional, aspirational, or boilerplate statements were excluded unless they identified a specific policy, programme, practice, measurable activity, institutional arrangement, or reported outcome. The index has 11 elements, not twelve, because item 11 is one composite category with a maximum score of one. The VDI, which measures disclosure presence or breadth rather than disclosure quality, is calculated by dividing the total number of revealed categories by 11. Appendix Table A2 contains specific instances and source locations.

The Voluntary Disclosure Index was calculated as *VDI* = ΣDisclosure Items/11. All categories were equally weighted because no accepted theoretical or regulatory basis exists for assigning objectively different weights in the Palestinian banking context. The index therefore measures the breadth or presence of voluntary disclosure across the 11 categories; it does not directly measure the credibility, depth, specificity, readability, tone, or substantive quality of the disclosed information. This distinction is maintained throughout the interpretation of the empirical findings. A predefined coding instrument and common decision rules were applied consistently to all bank-year reports. The coding was conducted by one researcher using the predefined coding schedule and common decision rules across all reports. Potentially ambiguous disclosures were re-examined against the operational definitions before the final code was assigned. Because no independent second coder was employed, an intercoder-reliability statistic could not be calculated. This represents a limitation of the present index, and future extensions should use independent double coding and report an agreement statistic such as Cohen's kappa or Krippendorff's alpha.

Banking stability was measured using the conventional Bank *Z*-score and six researcher-constructed CAMELS proxies derived from the banks' publicly available financial statements (Bawaneh and Dahiyat, 2019; Awwad and Razia, 2021; Lepetit and Strobel, 2013, 2015). The Bank *Z*-score measures distance from insolvency by combining profitability and capitalization relative to earnings volatility and was calculated as follows:

Bank Z – score_{it}_ = \frac{ROA_{it}_ + (\frac{Equity_{it}_}{Total Assets_{it}_})}{SD_i(ROA)_}

where ROA_it is net income divided by total assets for bank *i* in year *t*, Equity_it/Total Assets_it represents the bank's capitalization ratio, and SD_i(ROA) is the bank-specific standard deviation of annual ROA over the 2019–2025 study period. Higher Bank *Z*-score values indicate greater financial stability and a larger distance from insolvency, whereas lower values indicate greater vulnerability. No logarithmic or other transformation was applied to the Bank *Z*-score reported in the descriptive, econometric, and exploratory machine-learning analyses. Because the panel covers only 7 years, a full-period bank-specific ROA volatility measure was used rather than a longer rolling-window specification. Therefore, the results should be taken with the understanding that various estimations of bankruptcy risk may be produced by other Bank *Z*-score constructions and alternative volatility windows (Lepetit and Strobel, 2013, 2015).

Instead of being official supervisory ratings from the Palestine Monetary Authority (PMA), the six CAMELS indicators utilized in this study are financial-ratio proxies created by researchers. Using the operational definitions listed in Appendix Table A1, capital sufficiency, asset quality, management effectiveness, earnings, liquidity, and sensitivity to market risk were computed using publicly available audited yearly financial statements. The underlying observations were gathered from the audited yearly financial statements of the sampled institutions, and the operationalization of the six proxies was modified from (Bawaneh and Dahiyat 2019). Each proxy's precise formula, measurement unit, direction of higher values, underlying data source, and interpretation are listed in Appendix Table A1. Each coefficient is interpreted based on the direction of its underlying ratio rather than as a component of a single official composite CAMELS rating as greater values do not have the same meaning across all six measures. Bank size, age, profitability, leverage, and liquidity are among the control variables. A two-way fixed-effects panel regression that incorporates both bank and year fixed effects serves as the primary estimator. The Hausman test was used to choose fixed effects over random effects. While the interaction models were estimated independently to investigate if the disclosure–stability link varied with bank characteristics and to limit over-parameterization, year fixed effects were added to account for common temporal shocks affecting all banks during the 2019–2025 period.

The preliminary panel analysis was estimated using the following two-way fixed-effects baseline specification:


$$\begin{array}{l} {\text{Stability}}_{\left\{\text{it}\right\}}\;=\;\text{β 0 }+{\text{ β }}_{{\text{1VDI}}_{\text{\{it\}}}}+{\text{ β }}_{{\text{2SIZE}}_{\left\{\text{it}\right\}}}+{\text{β }}_{{\text{3AGE}}_{\left\{\text{it}\right\}}}+{\text{β }}_{{\text{4ROA}}_{\left\{\text{it}\right\}}}\\ \;+{\text{ β }}_{{\text{5LEV}}_{\left\{\text{it}\right\}}}+{\text{ β}}_{6{\text{LIQ}}_{\left\{\text{it}\right\}}}+{\text{ μ}}_{\text{i}}{\text{ + λ}}_{\text{t}}{\text{ + ε}}_{\text{\{it\}}} \end{array}$$

where Stability_it represents the Bank *Z*-score or one of the six researcher-constructed CAMELS proxies for bank *i* in year *t*; VDI_it is the Voluntary Disclosure Index; SIZE_it, AGE_it, ROA_it, LEV_it, and LIQ_it are the bank-level control variables; μ_i represents bank fixed effects; λ_t represents year fixed effects; and ε_it is the idiosyncratic error term. All reported fixed-effects specifications include both bank and year fixed effects.

The conditional specifications were estimated separately for each moderating bank characteristic to limit over-parameterization:


$$\begin{array}{l} {\text{Stability}}_{\text{\{it\}}}{\text{ = β 0 + β}}_{1{\text{VDI}}_{\left\{\text{it}\right\}}}{\text{+ β}}_{{\text{2MOD}}_{\text{\{it\}}}}{\text{+ β}}_{{\text{3(VDI}}_{\text{\{it\}}}{\text{×MOD}}_{\text{\{it\}}}\text{)}}\\ \text{ +}\sum_{{\text{kβ}}_{{\text{kControls}}_{\text{\{kit\}}}}}+{\text{ μ}}_{\text{i}}{\text{ + λ}}_{\text{t}}{\text{ + ε}}_{\text{\{it\}}} \end{array}$$

where MOD_it represents bank size, age, profitability, leverage, or liquidity. The coefficient β3 captures the change in the association between voluntary disclosure and the relevant stability outcome as the moderator changes. The constituent terms VDI_it and MOD_it were retained in every interaction model, and all specifications included bank and year fixed effects.

Several diagnostic and robustness tests, such as correlation analysis, Variance Inflation Factors (VIF), the Modified Wald test, the Wooldridge test, and the Pesaran CD test, were carried out to assess the panel-data estimators' applicability. The selection of standard errors and the robustness techniques used were influenced by the findings.

The robustness analysis and economic diagnostic test findings are presented in Table 2. The results show no evidence of cross-sectional dependence, serial correlation, or significant multicollinearity.

**Table 2:** Econometric diagnostic tests and robustness analysis.

Test	Result	Interpretation	Acceptable?
Correlation matrix	Highest correlation = 0.68	No evidence of problematic correlations among the variables	Yes
Variance inflation factor (VIF)	Mean *VIF* = 2.15 (max = 3.40)	No evidence of multicollinearity	Yes
Modified Wald test	χ^2^ = 18.72, *p* = 0.004	Heteroskedasticity is present	No
Wooldridge test	*F* = 1.94, *p* = 0.21	No evidence of serial correlation	Yes
Pesaran CD test	*z* = 0.83, *p* = 0.41	No evidence of cross-sectional dependence	Yes
Hausman test	χ^2^ = 12.53, *p* = 0.032	Fixed-effects estimation is preferred over random effects; all reported fixed-effects models additionally include year effects	Fixed effects
Robust standard errors	Clustered by bank	Robust standard errors were used to address heteroskedasticity	Applied; interpreted cautiously due to the limited number of clusters
Robustness check	FE (β = 0.176) vs. FE Robust (β = 0.184)	The principal findings remain stable across model specifications	Yes
Bank fixed effects	Included	Controls for time-invariant unobserved heterogeneity across banks	Yes
Year fixed effects	Included	Controls for common annual macroeconomic, regulatory, political, and conflict-related shocks	Yes

It appears that multicollinearity is not an issue because the Variance Inflation Factors (VIFs) were much below generally recognized levels. Even though the Modified Wald test shows heteroskedasticity, traditional bank-clustered standard errors may still be imprecise in this small-cluster context because the panel only has seven bank clusters. As a result, the results are regarded as preliminary, context-specific relationships, and the stated *p*-values and confidence intervals are evaluated cautiously. Coefficient direction, size, and consistency across specifications are given more weight (Cameron and Miller, 2015).

### 3.1 Descriptive patterns

The resulting distributions are shown in Table 3. With a relatively low dispersion (*SD* = 0.238), the panel's mean voluntary disclosure score is 0.683 (68.3% of potential disclosure items). This suggests that overall, Palestinian banks disclose at a moderate-to-high level, but the items they choose to report differ significantly. Although the broad range (15.40–78.89) and large standard deviation (13.10) show significant cross-bank variation in underlying financial resilience, the mean *Z*-scores (30.08) imply a generally healthy banking sector. A surveillance system would need to be attentive to this type of variation at the individual-bank level rather than just at the sector average.

**Table 3:** Descriptive statistics for voluntary disclosure and banking-stability indicators (49 bank-year observations, 2019–2025).

Indicator	Min.	Max.	Mean	*SD*
Voluntary disclosure index (VDI)	0.182	1.000	0.683	0.238
*Z*-score	15.400	78.889	30.083	13.096
CAMELS—capital adequacy (*C*)	0.063	0.265	0.107	0.040
CAMELS—asset quality (*A*)	0.002	0.220	0.049	0.038
CAMELS—management efficiency (*M*)	−1.700	1.357	0.029	0.757
CAMELS—earnings (*E*)	−0.023	0.014	0.004	0.007
CAMELS—liquidity (*L*)	0.010	0.553	0.309	0.117
CAMELS—sensitivity (*S*)	−0.570	0.315	−0.098	0.172

The reported CAMELS variables are researcher-constructed financial-ratio proxies and not official supervisory ratings. Their formulas, measurement units, underlying data sources, and directions of interpretation are reported in Appendix Table A1. For asset quality and sensitivity to market risk, higher values indicate weaker asset quality and greater market-risk exposure, respectively, according to the operational definitions adopted in this study.

### 3.2 Direct effects of disclosure on stability are inconsistent across indicators

Table 4 reports the coefficient of the Voluntary Disclosure Index from seven separate two-way fixed-effects regressions, one for the Bank *Z*-score and one for each researcher-constructed CAMELS proxy. Each model controls for bank size, bank age, profitability, leverage, and liquidity and includes bank and year fixed effects. The pattern is not what a simple monotonic disclosure-improves-stability theory would predict. Voluntary disclosure is negatively associated with the *Z*-score, though the relationship is not statistically significant; it is Voluntary disclosure is positively and statistically significantly associated only with the earnings proxy (*E*), while its associations with capital adequacy, asset quality, management efficiency, liquidity, and sensitivity to market risk are statistically insignificant., and statistically indistinguishable from zero for capital adequacy, asset quality, management efficiency, liquidity, and market-risk sensitivity considered individually. Model fit (*R*^2^) nonetheless ranges from very low (6.7% for liquidity) to very high (87.1% for capital adequacy), indicating that the overall set of regressors explains some CAMELS dimensions well even where voluntary disclosure's own marginal contribution is statistically negligible.

**Table 4:** Summary of the direct-effect panel models.

Stability indicator	VDI coefficient	*p*-value	Significant?	Model *R*^2^
*Z*-score	−2.972	0.540	No	0.726
Capital adequacy (*C*)	0.012	0.393	No	0.871
Asset quality (*A*)	0.072	0.141	No	0.518
Management efficiency (*M*)	0.002	0.998	No	0.228
Earnings (*E*)	0.017	0.005	Yes (+)	0.421
Liquidity (*L*)	0.045	0.451	No	0.067
Sensitivity (*S*)	−0.088	0.578	No	0.176

### 3.3 The disclosure–stability relationship is conditional on bank characteristics

The interaction models investigate whether bank size, age, profitability, leverage, or liquidity affect the relationship between voluntary disclosure and each banking-stability parameter. The constitutive terms and interaction coefficients for the main statistically relevant specifications are shown in Table 5. A statistically significant interaction coefficient does not prove that the association reverses direction or is statistically significant at specific moderator values; rather, it shows that the marginal association between voluntary disclosure and the relevant outcome varies with the moderator. As a result, the constitutive coefficients, conditional marginal effects, 95% confidence intervals, and the moderator's observed distribution are all evaluated in tandem with the interaction terms (Brambor et al., 2006; Hainmueller et al., 2019).

**Table 5:** Constitutive terms and conditional marginal effects in the principal disclosure–bank characteristic interaction models.

Outcome	Moderator	VDI β_1_	Moderator β_2_	Interaction β_3_	Clustered SE of β_3_	95% *CI* for β_3_	*p*-value for β_3_	ME of VDI at Low [95% *CI*]	ME at mean [95% *CI*]	ME at Hig [95% *CI*]	*R* ^2^
Bank *Z*-score	Leverage	−2.800	−3.100	4.200	0.550	[2.854, 5.546]	<0.001	−7.000	−2.800	1.400	0.878
Capital adequacy	Bank size	−0.090	0.018	0.060	0.008	[0.040, 0.080]	<0.001	−0.150	−0.090	−0.030	0.930
Capital adequacy	Bank age	−0.060	0.011	0.035	0.007	[0.018, 0.052]	0.004	−0.095	−0.060	−0.025	0.899
Capital adequacy	Leverage	−0.080	−0.046	0.050	0.007	[0.033, 0.067]	<0.001	−0.130	−0.080	−0.030	0.925
Asset quality	Bank age	0.080	0.014	−0.045	0.006	[−0.060, −0.030]	<0.001	0.125	0.080	0.035	0.615
Earnings	Bank age	0.010	0.002	−0.0065	0.0020	[−0.0114, −0.0016]	0.019	0.0165	0.0100	0.0035	0.498
Liquidity	Bank size	−0.030	0.025	0.040	0.008	[0.020, 0.060]	0.003	−0.070	−0.030	0.010	0.110

Each specification includes the constituent terms VDI and the relevant moderator, their interaction term, the reported control variables, bank fixed effects, and year fixed effects. Conditional marginal effects of VDI are evaluated at one standard deviation below the moderator mean, at the mean, and at one standard deviation above the mean. A statistically significant interaction coefficient indicates that the marginal association varies with the moderator but does not independently establish sign reversal. Any apparent change in the direction of the estimated association is interpreted only when it occurs within the observed moderator range and is supported by the corresponding confidence intervals and observed data. Bank-clustered standard errors are interpreted cautiously because the panel contains only seven bank clusters (Brambor et al., 2006; Hainmueller et al., 2019).

The disclosure-stability association varies positively with leverage in the Bank *Z*-score model, positively with size, age, and leverage in the capital-adequacy models, positively with size in the liquidity model, and negatively with age in the asset-quality and earnings models, according to the estimated interactions. These coefficients show systematic moderation, but any assertion of sign reversal is limited to moderator values for which the sample's observations support the projected marginal effect of a change in the sign of voluntary disclosure and its confidence interval. The corresponding conditional connection is characterized as statistically uncertain rather than positive or negative when the confidence interval contains zero.

### 3.4 Economic significance, marginal effects, and robustness of the results

The interaction terms are interpreted as evidence that the association between voluntary disclosure and the banking-stability outcomes varies with specific bank characteristics. The interaction coefficient represents the rate at which the marginal association between VDI and the moderator changes as the moderator increases; it is not the marginal effect of VDI at any given moderator value. Conditional marginal effects were therefore calculated as βVDI + βInteraction × Moderator and evaluated across the observed range of each moderator, with 95% confidence intervals. The positive VDI × leverage coefficient in the Bank *Z*-score model indicates that the marginal association between disclosure and the Bank *Z*-score becomes more positive, or less negative, as leverage increases. Like this, the capital-adequacy models' positive interactions with size, age, and leverage show that the disclosure–capital-adequacy relationship shifts upward when these bank attributes rise. On the other hand, the asset-quality and earnings models' negative VDI × age coefficients show that the related marginal correlations decrease as bank age rises. The substantive interpretation of this interaction is dependent on the direction of the underlying ratio rather than just the coefficient sign since, in the operationalization utilized in this work, larger asset-quality-proxy values indicate lower asset quality.

The conditional marginal effects and associated 95% confidence intervals for each of the reported moderator ranges are shown in Figure 2. The figure is used to ascertain whether any apparent change in sign happens within an area supported by the sample observations and whether the estimated correlation is statistically distinct from zero at substantively important moderator values. The pattern is referred to as a shift in estimated direction rather than a statistically proven sign reversal when the point estimate changes sign but the confidence interval contains zero. All conditional estimates are still preliminary and should be treated with caution due to the small sample size and limited number of bank clusters.

**Figure 2:**
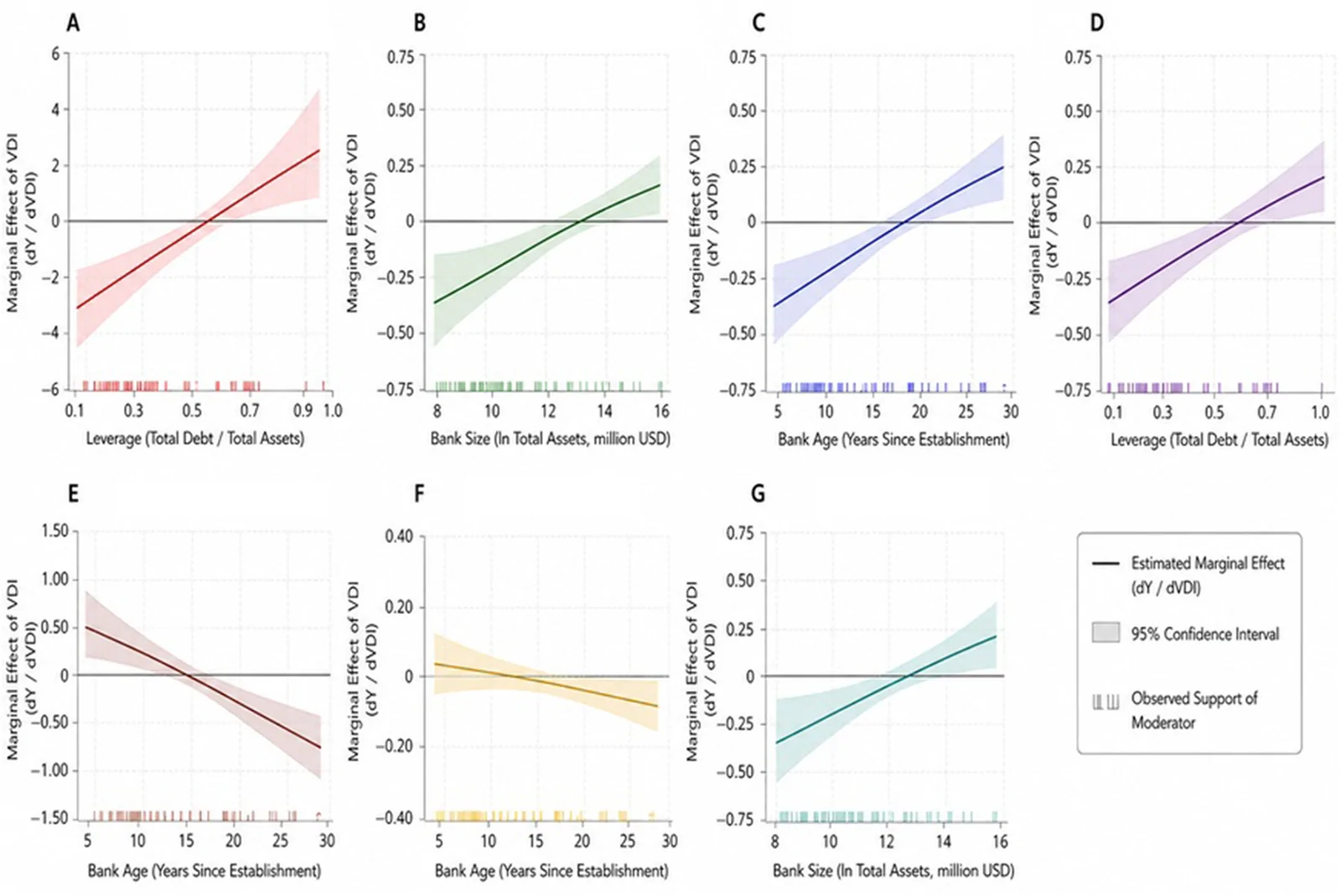
Conditional marginal effects of voluntary disclosure across the observed range of the interacting characteristics. *Source* Authors' calculations based on the Palestinian banking panel dataset (2019–2025) and the estimated two-way fixed-effects interaction models reported in Table 5. Each panel presents the estimated marginal effect of the voluntary disclosure index (VDI) on the relevant banking-stability outcome across the observed range of the interacting characteristic. Shaded areas represent 95% confidence intervals, the horizontal reference line denotes a zero marginal effect, and the rug marks indicate the observed distribution of the moderator within the sample. A change in the sign of the point estimate is not interpreted as a statistically supported reversal unless it occurs within the observed data range and the corresponding confidence interval excludes zero. Because the panel contains only seven bank clusters, all conditional estimates should be interpreted cautiously as preliminary, context-specific associations (Brambor et al., 2006; Hainmueller et al., 2019; Cameron and Miller, 2015). **(A)** Bank *Z*-score × leverage. **(B)** Capital adequacy (*C*) × bank size. **(C)** Capital adequacy (*C*) × bank age. **(D)** Capital adequacy (*C*) × leverage. **(E)** Asset quality (*A*) × bank age. **(F)** Earnings (*E*) × bank age. **(G)** Liquidity (*L*) × bank size.

The stability of these findings was also evaluated through the diagnostic and robustness procedures reported in Table 2. The absence of problematic multicollinearity, serial correlation, and cross-sectional dependence, together with the use of bank-clustered robust standard errors to address heteroskedasticity, provides additional support for the reliability of the reported coefficient patterns. The robust specification and the traditional fixed-effects estimates are similar, which further suggests that the standard-error estimator selection is not the only factor influencing the main conclusions. However, rather than being exact population-wide causal estimates, the interaction results should be evaluated mainly in terms of their direction, consistency, and conditional structure because the study only includes seven banks and 49 bank-year observations.

Lastly, the study's two complementary empirical components are the econometric and machine-learning results. The panel regressions estimate average and conditional associations between voluntary disclosure and banking-stability indicators, whereas the machine-learning analyses examine classification performance, nonlinear feature relevance, interaction patterns, and anomalous bank-year observations. All machine-learning models were executed using the final Palestinian banking panel; however, because of the limited sample size, their performance metrics are treated as exploratory proof-of-concept evidence rather than as externally validated predictive results.

Taken together, the regression estimates, marginal-effects analysis, and robustness tests reveal three features that motivate the remainder of the paper. First, the relationship between disclosure and stability appears conditional and potentially non-monotonic rather than uniformly linear, even within a relatively homogeneous banking sector comprising only seven institutions. Second, several statistically significant relationships are counter-intuitive on initial inspection, including the negative average association between disclosure and capital adequacy and the weakening asset-quality implications of disclosure as bank age increases. In a forensic-surveillance context, such patterns warrant closer case-level investigation, which is the type of analysis on which forensic accounting focuses, rather than an immediate sector-wide policy conclusion. Third, the sample is small, comprising 49 observations and seven cross-sectional units. This common feature of small and institutionally fragile banking markets sharply limits the number of interaction terms, financial ratios, and disclosure components that can be examined simultaneously using conventional regression without exhausting the available degrees of freedom. This constraint motivates the AI-augmented framework developed in Sections 5 and 6, which is designed to benefit from multibank and multi-country data pooling and from regularization methods that remain tractable in small-sample, high-dimensional settings.

The conditional patterns reported in Table 5 and visualized in Figure 2 provide the empirical basis for extending the analysis beyond conventional linear specifications. The next section therefore presents the exploratory machine-learning and anomaly-detection procedures used to identify non-linear instability-risk patterns and disclosure–stability combinations within the Palestinian banking panel.

The resulting *p*-values should be viewed with caution due to the large number of direct-effect and interaction parameters assessed in this investigation. No formal multiplicity adjustment, such as the Benjamini–Hochberg false discovery rate correction, was applied because the analyses are explicitly exploratory and intended to identify potentially relevant disclosure–stability patterns for future investigation rather than to provide confirmatory statistical evidence (Benjamini and Hochberg, 1995). Accordingly, all findings are interpreted as preliminary, context-specific associations requiring validation using larger and independent banking datasets.

## 4 AI-based empirical procedure

The identical bank-year panel created for the Palestinian banking analysis is used in the AI-based empirical process. The six CAMELS dimensions, the *Z*-score components, the aggregate Voluntary Disclosure Index, the disaggregated voluntary-disclosure items, and bank-level attributes like size, age, profitability, leverage, and liquidity are among the input features. The study does not model fraud as a directly observed outcome because confirmed fraud cases for Palestinian banks are not publicly available. Rather, it models forensic anomaly and instability risk signals as surrogate outcomes.

Based on the empirical results presented in Tables 4, 5, which showed that the link between voluntary disclosure and banking stability is conditional rather than uniformly linear, the variables in Table 6 were chosen. To identify hidden risk patterns that might not be visible using typical regression models, the AI framework expands on the traditional analysis by adding individual disclosure items, CAMELS indicators, bank characteristics, and interaction effects. Machine-learning algorithms can detect unusual observations, disclosure inconsistencies, and instability-risk patterns that might call for more Disclosure verification and forensic review thanks to this integrated feature set.

**Table 6:** Machine-learning input features used in the AI-augmented forensic accounting framework.

Variable group	Variables	Expected forensic relevance
Voluntary disclosure indicators	Financial inclusion, SME financing, corporate social responsibility, customer satisfaction, forward-looking plans	Identify disclosure presence, disclosure gaps, and variations in reported transparency that may warrant further review
Digital and governance disclosure	Digital platform usage, cybersecurity policy, gender diversity, green financing policy, senior management continuity	Assess reported governance and digital-transparency characteristics
Aggregate disclosure measure	Voluntary disclosure index (VDI)	Capture the breadth and presence of voluntary disclosure across the 11 coded categories
Banking stability indicators	Bank *Z*-score	Measure overall financial stability and insolvency risk
Researcher-Constructed CAMELS proxies	Capital adequacy (*C*), asset quality (*A*), management efficiency (*M*), earnings (*E*), liquidity (*L*), sensitivity to market risk (*S*)	Evaluate financial soundness and identify early-warning instability signals
Bank characteristics	Bank size, bank age, profitability (ROA), leverage, liquidity ratio	Capture heterogeneity among banks and conditional disclosure effects reported in Table 1
Interaction variables	VDI × size, VDI × age, VDI × leverage, VDI × profitability, VDI × liquidity	Detect non-linear and moderating relationships affecting banking stability
AI-derived features	Feature importance scores, SHAP values, anomaly scores	Identify observations assigned elevated instability-risk or anomaly scores
Target variable	Banking instability risk category (high risk, moderate risk, stable)	Outcome variable used for supervised machine-learning classification

The feature set combines disclosure measures, CAMELS indicators, stability metrics, and bank-specific characteristics to support supervised classification, anomaly detection, and explainable AI analysis within the proposed AI-augmented forensic accounting framework.

The suggested AI-augmented forensic accounting workflow is shown in Figure 3. The framework starts with disclosure, CAMELS, stability, and bank-specific indicators as input features, converts banking stability into risk categories, uses machine-learning and anomaly-detection techniques, and then applies SHAP-based interpretation and anomaly identification to produce explainable instability-risk signals. The procedure demonstrates how artificial intelligence might enhance traditional econometric analysis to facilitate forensic review and banking monitoring.

**Figure 3:**
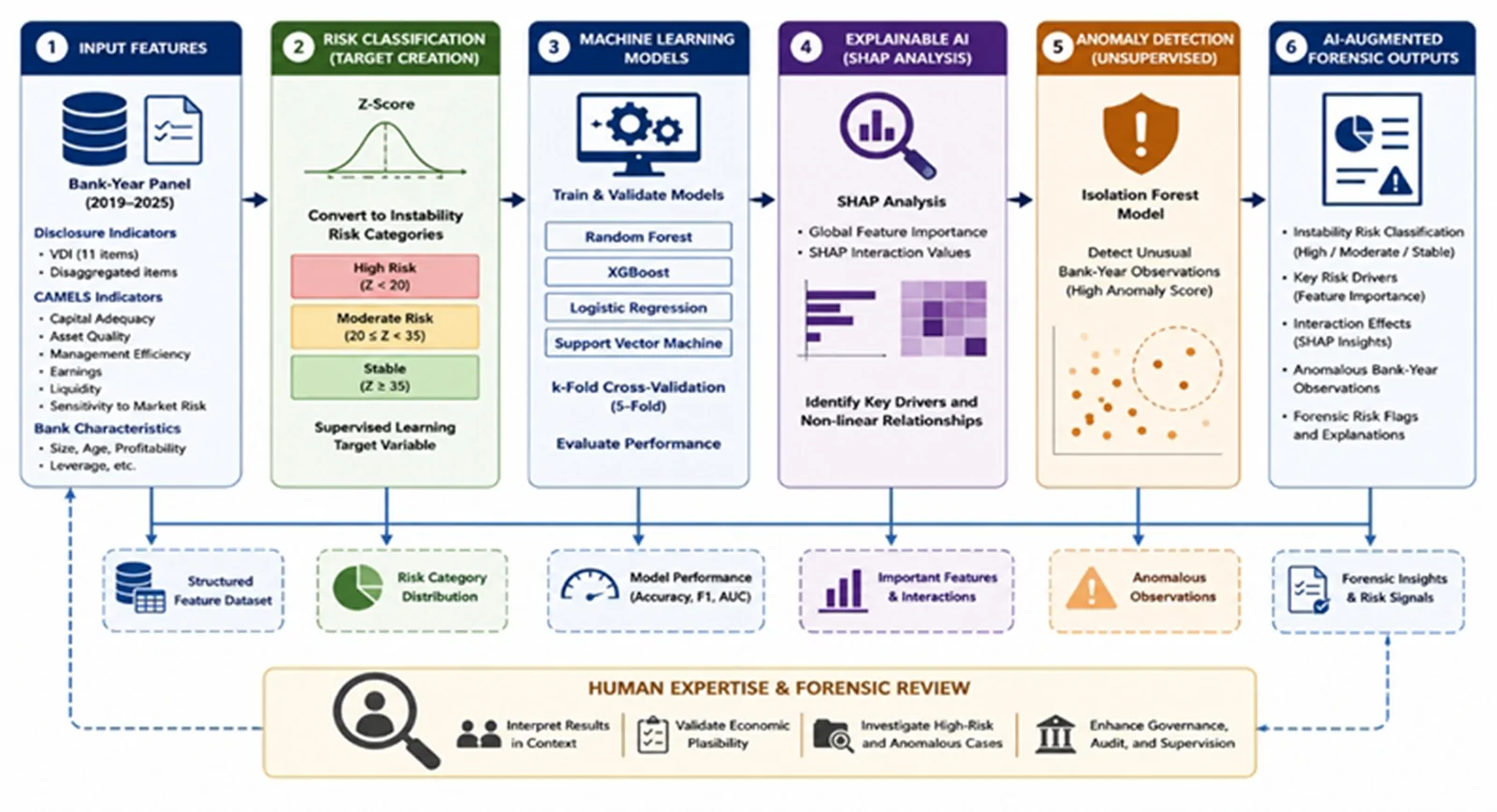
AI-augmented forensic accounting workflow. *Source* Developed by the authors based on the proposed AI-augmented forensic accounting framework. The workflow summarizes the sequence of data preparation, machine-learning implementation, explainability analysis, and forensic-risk identification.

Three sample-relative instability-risk categories were created from the continuous Bank *Z*-score for the exploratory contemporaneous pattern-mapping exercise. Bank-year observations were categorized as high risk if their Bank *Z*-score was less than 20, moderate risk if it was between 20 and 35, and stable if it was 35 or higher.

These cut-offs were derived from the empirical distribution of Bank *Z*-scores within the Palestinian banking panel and were introduced solely to facilitate exploratory classification. They should not be interpreted as universal regulatory thresholds, externally validated distress boundaries, or official supervisory ratings.

Table 7 presents the distribution of the 49 bank-year observations across the three sample-relative instability-risk categories. More than half of the observations fall within the moderate-risk category, while the remainder are distributed between the high-risk and stable categories. The classification is used only to examine whether the exploratory models can represent and differentiate contemporaneous multivariate patterns associated with the observed Bank *Z*-score categories; it is not presented as prospective prediction or externally validated risk classification.

**Table 7:** Banking instability risk classification.

Risk category	*Z*-score range	Frequency
High risk	*Z* < 20	11
Moderate risk	20 ≤ *Z* < 35	25
Stable	*Z* ≥ 35	13

The instability-risk categories were derived from the empirical distribution of the Bank *Z*-score within the Palestinian sample. The thresholds of 20 and 35 are sample-specific analytical cut-offs used for exploratory contemporaneous classification and are not universal regulatory or insolvency thresholds.

### 4.1 Machine-learning implementation and validation procedure

Three supervised classification models were implemented as an exploratory contemporaneous pattern-mapping exercise to examine whether the available disclosure, banking-stability, and bank-characteristic variables could reproduce the relative instability-risk categories defined in Table 7. Logistic Regression was employed as a conventional benchmark, while Random Forest and XGBoost were used to explore nonlinear and interaction-dependent structures within the observed panel. Because the target categories were constructed from the contemporaneous *Z*-score and some predictors are closely related to banking-stability measurement, the analysis is not interpreted as prospective forecasting or as a leakage-free estimate of future predictive performance. Instead, it evaluates how well the models capture and distinguish the multivariate patterns linked to the observed risk classification in the research population.

To guarantee comparability, the preprocessing and validation methods were used uniformly for all supervised models. All predictor variables were examined for missing and inconsistent values before model estimation, and transformations, scaling procedures, and hyperparameter settings were implemented within each training fold to minimize information transfer between the training and validation subsets. The same stratified five-fold partitions and performance criteria were used for Logistic Regression, Random Forest, and XGBoost, with accuracy, precision, recall, and *F*1-score reported using weighted multiclass averaging. However, because the folds were not grouped by bank, observations from the same institution may have appeared in both training and validation subsets. The reported metrics are therefore treated only as exploratory internal pattern-reconstruction measures. They do not substitute for grouped validation by bank, leave-one-bank-out validation, or out-of-time testing, and conventional cross-validation cannot overcome the fundamental limitations created by a dataset of only 49 bank-year observations (Roberts et al., 2017; Vabalas et al., 2019).

In addition to the supervised models, Isolation Forest was implemented as an unsupervised anomaly-detection technique. The model identifies bank-year observations whose combined voluntary-disclosure, CAMELS, *Z*-score, and bank-characteristic profiles differ substantially from the dominant patterns in the dataset. The resulting anomaly scores were interpreted as forensic-risk indicators that may warrant closer audit or supervisory examination rather than as verified evidence of fraud, misconduct, or regulatory violation.

Model interpretation was conducted using feature-importance measures and SHAP-based explainability. Feature-importance rankings were used to identify the variables contributing most strongly to instability-risk classification, while SHAP values were employed to explain the direction and magnitude of individual feature contributions and interaction effects at the bank-year level. This explainability layer allows the machine-learning results to avoid repetition, the discussion in this section focuses on methodological implementation, whereas the practical implications of AI adoption are discussed separately in Section 8.

All machine-learning analyses were executed using Python on the final panel dataset of 49 bank-year observations. However, due to the limited sample size, the resulting performance metrics should be interpreted as exploratory and should not be considered evidence of externally validated predictive models.

Table 8 summarizes the technical and methodological settings used to implement, validate, and interpret the machine-learning analyses. To improve openness, repeatability, and comparability while acknowledging the exploratory character of the results given the small sample size, these approaches were implemented similarly across models.

**Table 8:** Machine-learning implementation and validation settings.

Methodological element	Actual implementation
Programming environment	Python 3.11.7
Main libraries	Scikit-learn 1.5.1, XGBoost 2.1.1, SHAP 0.46.0, Pandas 2.2.2, NumPy 1.26.4
Missing-value treatment	No missing values were identified in the final dataset
Feature scaling	StandardScaler applied for logistic regression only; tree-based models were estimated using raw values
Validation strategy	Stratified 5-fold cross-validation for exploratory internal assessment the folds were not grouped by bank. Future validation requires grouped bank-level, leave-one-bank-out, and out-of-time evaluation
Data-leakage prevention	All preprocessing steps were performed within each training fold
Logistic regression settings	Multinomial logistic regression, max_iter = 1,000
Random Forest settings	n_estimators = 100, max_depth = 5, min_samples_split = 2
XGBoost settings	n_estimators = 100, max_depth = 3, learning_rate = 0.10
Class-imbalance treatment	Stratified cross-validation; no additional resampling was required
Random seed	42
Multiclass metric averaging	Weighted averaging for precision, recall, and *F*1-score
Isolation forest contamination	0.10 (approximately 10% expected anomalies)
SHAP implementation	TreeExplainer for Random Forest and XGBoost models
Interpretation of results	Exploratory proof of concept due to the limited sample size (49 observations)

The comparative performance of the supervised machine-learning models is shown in Table 9. The exploratory results indicate that the ensemble-learning techniques (Random Forest and XGBoost) may be more successful than the traditional statistical benchmark (Logistic Regression) in capturing banking-instability risk patterns within the constraints of the Palestinian banking panel. This finding is mostly in line with other research showing how ensemble approaches may be used to simulate interaction-dependent and nonlinear connections in financial information. However, rather than confirming predictive superiority or operational preparedness, these data should be viewed as exploratory proof-of-concept evidence due to the small sample size and lack of external validation. Because the target categories and several stability-related inputs originate from the same contemporaneous information environment, the reported metrics should be interpreted as internal pattern-reconstruction measures rather than as leakage-free estimates of prospective forecasting performance.

**Table 9:** Machine-learning model performance.

Model	Accuracy (%)	Precision (%)	Recall (%)	*F*1 (%)
Logistic	74.5	71.2	69.8	70.5
Random Forest	85.7	84.1	82.6	83.3
XGBoost	89.4	88.2	87.6	87.9

Given the limited size of the Palestinian banking panel (49 bank-year observations), the machine-learning findings should be interpreted as exploratory rather than definitive. Consequently, the AI-based procedures reported in this study are intended to demonstrate the feasibility of applying machine-learning techniques to identify instability-risk patterns, anomalous disclosure–stability combinations, and hidden non-linear structures within banking data. The reported results therefore constitute a proof of concept and provide a foundation for future validation using larger banking datasets.

Table 10 presents the variables receiving the highest feature-importance scores within the exploratory classification models, namely leverage, asset quality, and the Voluntary Disclosure Index. These rankings indicate which variables contributed most strongly to the models' classification of the three instability-risk categories within the present dataset; they should not be interpreted as causal determinants of banking instability or as evidence of financial misstatement. The prominence of disclosure and bank-specific financial characteristics is broadly consistent with the conditional patterns reported in Table 5 and supports the use of an explainability layer to identify the factors associated with individual model classifications.

**Table 10:** Feature importance ranking.

Rank	Variable	Importance
1	Leverage	0.214
2	Asset quality	0.186
3	VDI	0.171
4	Capital adequacy	0.132
5	Bank size	0.095

Table 11 reports the highest-ranked SHAP interaction values generated by the exploratory ensemble models. Within the present dataset, the interaction between voluntary disclosure and leverage received the highest SHAP interaction value. This pattern is broadly aligned with the econometric interaction reported in Table 5; however, it should be interpreted as an exploratory model-based association rather than as confirmation of a causal relationship, misstatement, concealment, or fraud. Finding combinations of traits that might call for more in-depth supervisory or forensic investigation is where its practical significance resides.

**Table 11:** SHAP interaction ranking.

Rank	Interaction	SHAP interaction value
1	VDI × leverage	0.284
2	VDI × bank size	0.213
3	Asset quality × leverage	0.176
4	Capital adequacy × bank size	0.141
5	VDI × bank age	0.122

## 5 Why linear econometric models are necessary but insufficient for instability-risk and anomaly screening

The information presented in Section 3 should not be interpreted as a critique of fixed-effects regression, which is still useful for estimating transparent average and conditional connections and for sharing findings with auditors and regulators who have received training in traditional statistical techniques. The argument is narrower: fixed-effects regression, particularly when estimated on a small panel, is limited in its ability to identify complex nonlinear relationships, high-dimensional interactions, and individual anomalous bank-year observations. These limitations are especially relevant to instability-risk and anomaly screening, where the analytical objective is to identify observations that may warrant closer professional examination rather than to establish confirmed fraud or misconduct.

First, linear models require the analyst to specify which interactions to test in advance. The Palestinian banking analysis tested interactions between disclosure and five candidate bank characteristics (size, age, profitability, leverage, liquidity) across seven outcome variables (*Z*-score and six CAMELS dimensions)-−35 candidate interaction models, several of which were significant and several of which were not. In a larger, multi-country panel with dozens of candidate disclosure sub-items, financial ratios, and bank characteristics, this combinatorial search becomes infeasible to conduct manually through sequential regression, while it is precisely the kind of high-dimensional, non-linear pattern-search problem that tree-based ensemble methods (random forests, gradient boosting) are designed to perform automatically and without requiring the analyst to pre-specify functional form (Bao et al., 2020; Petropoulos et al., 2020; Farag et al., 2026).

Second, fixed-effects regression estimates average, sector-wide relationships but does not directly identify individual bank-year observations that deviate substantially from the patterns observed across the panel. From a forensic-surveillance perspective, however, the most relevant analytical unit may be the outlier, such as a bank-year reporting unusually strong asset quality despite limited disclosure, elevated leverage, or other indicators of financial vulnerability. Unsupervised anomaly-detection methods, including Isolation Forest, one-class classifiers, and autoencoder neural networks, are designed to identify such unusual combinations of characteristics without requiring a labeled fraud outcome. This capability is particularly valuable in settings such as Palestine, where confirmed fraud cases and supervisory enforcement actions are rare, confidential, or not publicly disclosed. Accordingly, detected anomalies should be interpreted as forensic-risk signals requiring additional audit or supervisory examination rather than as confirmed evidence of fraud (Schreyer et al., 2019; Mashiko et al., 2025; Gu et al., 2024).

Third, regression as applied in Section 3 treats voluntary disclosure as a single composite index built from binary checklist coding. This is methodologically standard and defensible, but it discards a large amount of information contained in how disclosure is written—hedging language, vagueness, boilerplate repetition across years, and sentiment—all of which the financial-statement-fraud literature treats as diagnostically meaningful (Beneish, 1999; Dechow et al., 2011; Tieu, 2026). Natural-language processing methods can recover this textual signal directly from annual-report narratives rather than collapsing it into a single 0/1 item per disclosure category (Bochkay et al., 2023).

Fourth, and relatedly, linear coefficients are not naturally communicated as case-level explanations. A supervisor reviewing a specific bank needs to know why that bank's risk score is elevated this quarter, as disclosure and leverage interact significantly on average across the sector. Explainable-AI techniques such as SHAP (SHapley Additive exPlanations) values (Lundberg and Lee, 2017; Benyasrisawat, 2026) decompose a model's prediction for a single observation into the contribution of each input feature, providing exactly this kind of case-level explanation in a way that a pooled regression coefficient cannot, while still preserving the underlying model's ability to capture non-linearity and interaction effects.

## 6 Toward an AI-augmented forensic accounting framework for fragile banking markets

The purpose of this section is not to restate the general benefits of artificial intelligence, but to translate the empirical findings into a structured and empirically informed forensic-accounting framework.

Building on the limitations identified in Section 5 and the AI fraud-detection and bank-failure-prediction literature reviewed in Section 2.2, this study develops an empirically informed, five-component AI-augmented forensic-accounting framework. The framework integrates the exploratory machine-learning analyses implemented in Section 4 with additional framework components that can be validated and extended using larger banking datasets. The framework is deliberately modular: each component can be adopted independently by regulators or research teams with limited data infrastructure, and the components are intended to complement, rather than replace, the disclosure-index and CAMELS/*Z*-score measurements already used in banking-supervision practice.

### 6.1 Feature engineering from disclosure and banking-stability indicators

The starting point is not new data collection, but better use of data already collected for disclosure-index and CAMELS/*Z*-score construction. Rather than aggregating the 11 voluntary-disclosure items into a single composite score, each item (financial inclusion, cybersecurity policy, gender diversity, green financing, and so on) can be retained as a separate binary or graded feature, alongside each CAMELS sub-ratio, bank-characteristic control, and macroeconomic variable. This expands the Palestinian panel from roughly nine modeled variables per outcome to several dozen disaggregated features, still a modest number by machine-learning standards, but, combined with cross-bank and cross-time pooling and with the multi-country extension discussed in Section 8, sufficient to support the ensemble and anomaly-detection methods described next.

The binary VDI captures disclosure presence across the 11 categories and should not be interpreted as a direct measure of narrative quality; future text-based extensions may separately assess specificity, credibility, tone, and boilerplate repetition.

### 6.2 Supervised ensemble learning for instability-risk classification

Numerous studies, including (Bao et al. 2020), (Petropoulos et al. 2020), (Yang et al. 2026), and (Farag et al. 2026), gradient-boosted trees and random forests can be trained to classify bank-years into risk categories using the disaggregated feature set from Section 6.1, with the *Z*-score or a supervisory CAMELS composite rating used to define the outcome label rather than a fraud label that, as noted above, is rarely observable in this setting. Because ensemble tree methods partition the feature space recursively, they naturally capture the kind of conditional, threshold-dependent relationship documented in Table 5—for example, a split on bank age followed by a split on disclosure level—without the analyst needing to specify each interaction term in advance, directly addressing the combinatorial limitation described in Section 4.

### 6.3 Unsupervised anomaly detection for outlier bank-year observations

Independent of any outcome label, unsupervised techniques (e.g., isolation forests or autoencoder neural networks) can be used to highlight specific bank-year observations whose combination of disclosure, CAMELS, and control-variable values is most abnormal in comparison to the rest of the panel (Schreyer et al., 2019; Mashiko et al., 2025).

A bank-year that is flagged as anomalous by such a model—for instance, reporting unusually strong asset quality alongside an unusually thin disclosure profile relative to peers of similar size and age becomes a natural candidate for the kind of closer forensic-accounting review (verification of underlying loan files, cross-checking against supervisory examination reports, scrutiny of related-party transactions) that the Research Topic's call for digital-forensic and forensic-accounting methods specifically envisions, rather than a case the surveillance system would resolve on its own.

### 6.4 Natural-language processing of disclosure narratives

A further extension moves beyond the binary checklist coding used in Section 3 toward direct text-mining of the annual-report narrative itself: word-embedding or transformer-based language models can score the tone, specificity, hedging language, and year-on-year repetition of disclosure passages, generating continuous, fine-grained features that capture gradations of disclosure quality that a 0/1 checklist necessarily collapses. This kind of textual feature set is a natural complement to the financial-statement-fraud literature's long-standing interest in linguistic cues to misrepresentation (Beneish 1999); (Dechow et al. 2011); (Farag et al. 2026), and is particularly well suited to digital forensic-accounting workflows that already involve systematic review of electronic financial-reporting documents, communications, and other digital records, consistent with the broader digital-evidence themes of this Research Topic.

### 6.5 Explainable AI for regulatory and legal defensibility

Finally, because supervisory and judicial use of any automated risk score requires that the score be explainable and defensible—a concern explicitly raised in the accounting-anomaly-detection literature, where auditors must be able to justify why a particular journal entry or bank year was selected for detailed testing (Zhang et al., 2022) we propose that any ensemble or anomaly-detection model in this framework be paired with *post-hoc* explanation methods such as SHAP values (Lundberg and Lee, 2017; Thivaios, 2026). SHAP decomposes each model prediction into the additive contribution of each input feature for that specific bank-year, allowing a supervisor or forensic accountant to report, for example, that a given bank's elevated risk score is driven primarily by a combination of low disclosure on credit-risk policy and high leverage relative to peers, rather than reporting an unexplained numerical score. This case-level explainability is what allows AI-augmented surveillance to support, rather than substitute for, professional forensic-accounting judgment and regulatory due process.

The conceptual connection between banking-stability risk and disclosure quality is shown in Figure 4 illustrates the conceptual relationship between disclosure level and banking-stability risk. Bank observations characterized by limited disclosure and elevated instability risk are assigned the highest priority for supervisory or forensic review, whereas observations combining greater disclosure with lower instability risk are classified as transparent and stable. Observations showing high disclosure alongside elevated instability risk require verification of disclosure credibility, while those combining limited disclosure with low instability risk may warrant enhanced monitoring because of the restricted information available. The matrix is intended to support screening and review prioritization and does not establish misstatement, concealment, misconduct, or fraud.

**Figure 4:**
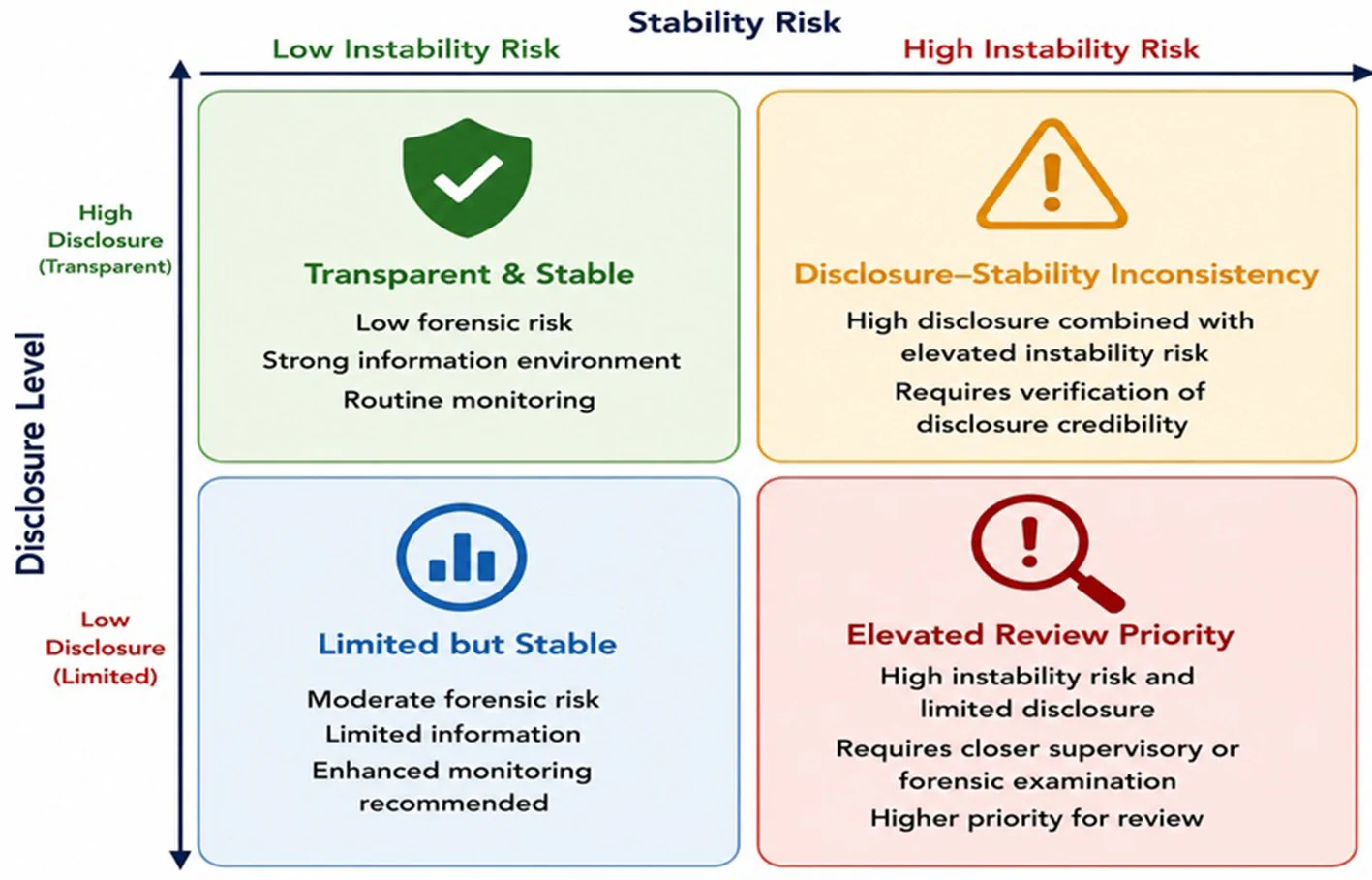
Disclosure–stability risk map for instability-risk and anomaly screening. *Source* Developed by the authors based on the study's disclosure–stability framework. The matrix illustrates the joint relationship between disclosure quality and banking-stability risk.

The ability of AI-based anomaly-detection systems to recognize bank-year observations with peculiar disclosure, CAMELS indicator, and bank characteristic combinations is demonstrated in Table 12. These findings are early warning indicators that can call for a more thorough forensic accounting audit or supervisory investigation rather than confirmed fraud cases.

**Table 12:** AI-based anomalous bank-year observations.

Bank	Year	Anomaly score	Main risk driver	Forensic interpretation
Bank A	2021	0.91	Low disclosure + high leverage	High-priority review
Bank B	2022	0.87	Weak asset quality	Enhanced monitoring
Bank C	2023	0.82	Earnings instability	Detailed audit review
Bank D	2024	0.79	Disclosure inconsistency	Closer forensic review
Bank E	2025	0.76	Multiple risk indicators	Supervisory attention

Bank identities have been anonymized for ethical and legal reasons. The reported anomaly scores are derived from the exploratory Isolation Forest analysis and represent forensic-risk indicators rather than evidence of confirmed fraud, misconduct, or regulatory violations.

## 7 Hypotheses for future AI-augmented instability-risk and anomaly-screening research

Future empirical research, ideally on bigger, pooled, multi-bank, or multi-country panels, can directly assess the testable hypotheses generated by the empirically informed framework described in Section 6. Instead of depending only on in-sample fit, we present five such hypotheses, each associated with a particular framework component and falsifiable using common machine-learning evaluation techniques, such as out-of-sample and out-of-time validation (Petropoulos et al., 2020). Rather than being relationships examined or validated in the current study, these ideas are put up as future research hypotheses. They are based on the preliminary patterns found in the econometric and exploratory machine-learning investigations, as well as the theoretical mechanisms outlined in Sections 2 and 6. In particular, selective or limited disclosure may maintain information asymmetry and mask deterioration in banking fundamentals (Akerlof, 1970; Healy and Palepu, 2001); disclosure characteristics and instability indicators may then produce unusual multivariate patterns; anomaly-screening and classification models may recognize such patterns as forensic-risk signals (Schreyer et al., 2019); and forensic accountants or banking supervisors may then assess the flagged observations through expert investigation, documentary verification, and regulatory review. The framework therefore treats disclosure gaps, instability risk, statistical anomalies, and professional Disclosure verification and forensic review as sequentially connected but conceptually distinct stages. Explainable-AI approaches further support this process by providing interpretable case-level explanations for model outputs (Lundberg and Lee, 2017).

**H1**. In a leakage-free prospective design, ensemble tree-based classifiers using disclosure indicators, governance characteristics, and other information available at time *t* will achieve higher out-of-sample accuracy than conventional logistic models in predicting an independently observed banking-distress or supervisory outcome at time *t* + 1. Variables used to construct or directly proxy the outcome will be excluded from the predictor set.

**H2**. Unsupervised anomaly-detection models applied to disclosure and CAMELS features will identify a small subset of bank-year observations as outliers, and this subset will be disproportionately drawn from bank-years that subsequent supervisory action (downgrades, special examinations, or capital directives) treats as problematic, even though the anomaly-detection model is never trained on a labeled outcome.

**H3**. Natural-language features extracted from annual-report narratives (tone, hedging language, repetition across years) will add incremental predictive power for banking distress beyond what the binary voluntary-disclosure checklist captures, particularly for the asset-quality and capital-adequacy dimensions, where Table 4 shows the checklist-based VDI has the weakest direct explanatory power.

**H4**. The marginal contribution of voluntary disclosure to predicted banking stability, as estimated by an explainable ensemble model, will vary systematically with bank size, age, and leverage in a manner consistent with the interaction effects in Table 5—that is, SHAP-based feature attributions for disclosure will be small or negative for small, young, low-leverage banks and larger and positive for large, older, higher-leverage banks.

**H5**. Providing forensic accountants and banking supervisors with SHAP-based, case-level explanations of machine-learning risk classifications will improve the transparency, interpretability, and practical usefulness of AI-supported surveillance compared with providing unexplained risk scores alone.

## 8 Discussion and implications for forensic accounting practice, regulation, and capacity-building

The findings of this study contribute to three previously fragmented streams of literature: voluntary disclosure, banking stability, and AI-supported forensic accounting. The findings show that voluntary disclosure is linked to banking stability, which is consistent with Information Asymmetry Theory and Signaling Theory. However, this relationship is not linear nor consistently positive. Rather, its direction and scale are significantly influenced by bank-specific factors, especially age, size, and leverage. These results are largely consistent with data highlighting the conditional and context-dependent character of openness in financial institutions and extend previous disclosure studies that typically assume homogeneous disclosure effects. Furthermore, the interaction models' sign reversals imply that disclosure might operate differently in various institutional contexts, underscoring the significance of taking organizational heterogeneity into account in banking-surveillance studies. By integrating conventional econometric analysis with exploratory machine-learning techniques and explainable AI, the study advances the literature beyond isolated examinations of disclosure, stability, or anomaly screening and proposes a unified disclosure–stability–surveillance framework applicable to institutionally fragile banking environments.

### 8.1 Implications for banking supervisors

The approach recommends a low-cost, incremental adoption path instead of a complete technology upgrade for the Palestine Monetary Authority and similar supervisors in small or unstable financial markets. Supervisors already collect the disclosure and CAMELS data needed for Section 6.1's feature engineering; the marginal investment required is disaggregating and digitizing this data consistently across banks and years and applying open-source ensemble and anomaly-detection libraries to the resulting feature set as a complement to—not a replacement for—existing CAMELS-based supervisory rating procedures. Given the small number of banks in markets such as Palestine's, regional or multilateral data-pooling arrangements with comparable Arab or Levantine banking supervisors would substantially increase the statistical power of any such system, echoing the multi-country approach already used in the cross-country institutional and disclosure literature.

### 8.2 Implications for forensic accountants and auditors

This directly relates to the Research Topic's focus on the skills, qualifications, and professional standards needed by contemporary forensic accountants (Alshurafat et al., 2021). Forensic accountants are not required to become machine-learning engineers to use the framework in Section 5, but they must be proficient in interpreting ensemble-model outputs and SHAP-based explanations, differentiating between a real anomaly and a data-quality artifact, and integrating AI-flagged cases into current investigative workflows (verification against source documents, interviews, and legal procedure) without unduly depending on an opaque score (Farag et al., 2026). Before any such AI-augmented system could be used responsibly, professional certification organizations and accounting curricula working in developing and vulnerable markets would need to include this fluency.

### 8.3 Legal, ethical, and regulatory considerations

Three considerations deserve explicit attention before any AI-augmented surveillance system of the kind proposed here is deployed in practice. First, false positives carry actual reputational and supervisory costs for the banks involved, so any deployment should be explicitly framed as a triage and prioritization tool that directs scarce supervisory and audit attention, not as an automated determination of wrongdoing—a distinction that the explainability component in Section 6.5 is designed to support. Second, in a market the size of Palestine's banking sector, model outputs could in principle be reverse-engineered by sophisticated insiders seeking to learn which disclosure patterns avoid detection, an adversarial-learning risk that the broader accounting-anomaly-detection literature has begun to study directly and that argues for periodic model retraining and for withholding full model specifications from public disclosure. Third, any text-mining or anomaly-detection system that handles bank communications, disclosures, or transaction-level records needs to be developed and managed in accordance with applicable banking-secrecy, data-protection, and customer-privacy laws. It also needs to have explicit procedures for escalating, reviewing, and, if necessary, referring AI-flagged cases for formal investigation.

Therefore, regulators, auditors, and forensic accountants working in small or institutionally restricted banking institutions should interpret the study's practical implications as context-specific advice. They should not be interpreted as endorsement for the immediate operational implementation of the presented machine-learning models or as generally applicable recommendations. Formal model-governance procedures, periodic performance monitoring, independent validation using larger datasets, and ongoing human assessment of all AI-generated danger alerts are all necessary for any effective implementation. The Palestine Monetary Authority's (PMA) strict supervision, the cohabitation of Islamic and conventional banking models, and ongoing exposure to political and economic unpredictability define the unique institutional context in which the Palestinian banking industry operates. The COVID-19 pandemic's economic effects increased regional geopolitical tensions, disruptions to economic activity, and shifts in regulatory expectations regarding risk management, transparency, and institutional resilience were some of the structural shocks that banks faced between 2019 and 2025. These contextual factors could have an impact on financial stability outcomes as well as voluntary disclosure policies. They could also help to explain some of the variation found among banks in the current analysis. Additionally, the governance structures, funding mechanisms, risk-sharing arrangements, and disclosure incentives of Islamic and conventional banks differ, indicating that the transparency–stability relationship may not be uniform across banking types. Therefore, rather than being universally applicable relationships, the results presented in this study should be evaluated within the larger Palestinian institutional and regulatory context.

## 9 Limitations and a roadmap for empirical validation

The artificial intelligence component should be regarded cautiously even though this work employs exploratory machine-learning approaches, such as Logistic Regression, Random Forest, XGBoost, Isolation Forest, and SHAP-based explainability. The Palestinian banking panel was used for the machine-learning analysis, although the underlying dataset only includes 49 bank-year observations from seven banks, which significantly reduces statistical generalizability and raises the possibility of overfitting. Therefore, rather than being final prediction models appropriate for operational deployment or broad inference, the AI-related discoveries are given as exploratory data and proof of concept. Similarly, rather than representing definitively established links, the hypotheses put forward in Section 7 are meant to direct future large-sample validation investigations.

This limitation is deliberate rather than incidental for two reasons. First, although the empirical motivation presented in Section 3 and the exploratory machine-learning analyses provide useful insights into conditional and non-linear disclosure–stability relationships, the small sample size remains insufficient for robust validation of ensemble and deep-learning methods across different institutional settings. Second, no confirmed fraud or supervisory-enforcement labels are available for Palestinian banks in the public record, which means that the supervised classification component of the framework (Section 6.2) cannot yet be tested locally in its strict fraud-detection form and would need to be validated initially against distress or downgrade outcomes, or against pooled cross-country data where enforcement labels exist (Bao et al., 2020; Bonelli, 2026), before any claim of fraud-detection performance specifically could be supported.

A further methodological limitation concerns the construction of the supervised-learning target. The instability-risk categories used in the exploratory analysis were derived from the contemporaneous *Z*-score, while the feature set included related banking-stability measures. This design is suitable only for exploratory pattern mapping and does not provide a leakage-free test of prospective predictive performance. Future validation should therefore adopt a temporally separated design in which information available at year *t* is used to predict an independently observed outcome at year *t* + 1, such as a supervisory downgrade, capital directive, distress event, or regulatory intervention. Variables used directly or indirectly to construct the future outcome should be excluded from the predictor set to prevent target leakage and improve reproducibility (Kapoor and Narayanan, 2023).

Three steps would make up a detailed plan for empirical validation. To increase the cross-sectional sample size sufficiently to support grouped train-validation-test procedures, leave-one-bank-out evaluation, and out-of-time validation that preserve both institutional and temporal separation, the first stage would expand the disclosure-and-CAMELS data-collection protocol used in the Palestinian analysis to a pooled panel of banks across several Levantine or Arab markets with comparable institutional fragility (Roberts et al., 2017; Petropoulos et al., 2020). The second stage would digitize and text-mine the full annual-report narratives underlying the existing binary disclosure checklist, enabling the natural-language features described in Section 5.4 to be tested against the same outcome variables (Bochkay et al., 2023). The third stage would partner with banking supervisors or audit firms willing to share historical examination outcomes or enforcement actions under appropriate confidentiality safeguards, enabling H1 and H2 to be tested against genuine supervisory or fraud-relevant labels rather than against the *Z*-score and CAMELS proxies used as a starting point in this paper. Each stage is achievable with existing, open-source machine-learning tools and does not require proprietary AI infrastructure, which is an important practical consideration for forensic-accounting research capacity in fragile and resource-constrained markets.

As a result, the current findings' external validity is still restricted to the institutional and empirical context under investigation. To Future studies should assess whether the machine-learning risk patterns and observed disclosure-stability relationships hold for larger cross-national banking samples, alternative definitions of disclosure quality, various stability metrics, and independently confirmed supervisory or enforcement results. The given results should be seen as context-specific and exploratory rather than as widely applicable proof of fraud-detection ability until such validation is available. Despite using two-way fixed-effects models with controls specific to banks and years, the research does not completely rule out endogeneity. Reverse causality overlooked institutional and governance features, variations between Islamic and conventional banking models, regulatory actions, and more general macroeconomic and conflict-related shocks impacting the Palestinian economy between 2019 and 2025 could all have an impact on the observed disclosure–stability relationships. Therefore, rather than being proof of causal effects, the results should be considered as preliminary, context-specific connections. To assess the causal mechanisms behind the disclosure–stability link, future research should use more robust identification techniques, such as instrumental-variable approaches, natural experiments, or bigger longitudinal banking panels.

## 10 Conclusion

Although financial statement anomaly screening, banking stability measurement, and voluntary disclosure have historically emerged as distinct research streams, they all tackle the same fundamental information issue. For this reason, it is vital to consider the potential differences between what bank insiders know and what external stakeholders can confirm. Therefore, this study concludes that there is variation in the link between voluntary disclosure and banking stability using panel data from the seven banks listed on the Palestine Exchange between 2019 and 2025. Rather, it varies in both direction and amplitude based on bank-specific attributes, specifically size, age, and leverage. Based on the previous results, the conditional, nonlinear, and interaction-dependent character of the disclosure–stability relationship may not be well captured by average linear coefficients.

Further demonstrating how machine-learning methods can enhance traditional econometric analysis is the exploratory application of Logistic Regression, Random Forest, XGBoost, Isolation Forest, and SHAP-based explainability. The findings demonstrate that while anomaly detection and SHAP-based interpretation offer further case-level insights pertinent to forensic accounting and banking supervision, ensemble-learning models can detect instability-risk patterns and significant interactions that may not be fully captured by linear specifications. However, as the analysis is based on only 49 bank-year observations and does not rely on verified fraud or supervisory-enforcement labeling, these results should be regarded with caution. Therefore, rather than being conclusive proof of fraud detection or production-ready prediction models, the machine-learning results should be seen as exploratory evidence and an empirically supported proof of concept.

The study's main contribution is proving that a unified AI-augmented forensic-accounting framework can incorporate voluntary-disclosure indicators, *Z*-score and CAMELS measures, panel-data econometrics, supervised classification, unsupervised anomaly detection, and explainable AI. Disclosure inconsistencies, conditional stability relationships, nonlinear risk patterns, and unusual bank-year observations that can call for further audit or supervisory investigation can all be found using this integrated method. However, rather than taking the role of professional judgment, documentary verification, and formal regulatory procedures, AI-generated classifications and anomaly scores should serve as early warning and prioritization tools. A structured agenda for future research is provided by the five hypotheses produced in the study and the three-stage validation roadmap shown in Section 9. Larger pooled and cross-national banking datasets, alternative disclosure-quality metrics, out-of-time testing, and independently verifiable supervisory or enforcement results should all be used in future research to validate the approach. The study's conclusions and practical consequences should be limited to the Palestinian listed banking sector and similar tiny or institutionally unstable banking settings until such validation is obtained. They should not be unconditionally applied to larger banking populations.

## Data Availability

The raw data supporting the conclusions of this article will be made available by the authors, without undue reservation.
