# Supplementary material for: Voluntary disclosure, banking stability, and AI-augmented forensic accounting: an exploratory econometric and machine-learning study of Palestinian banks
Source: Front Big Data. 2026 Sep 14;9:1914133. doi: 10.3389/fdata.2026.1914133 (PMC13616643; doi:10.3389/fdata.2026.1914133)
Supplement: Supplementary file 1 [file Supplementary_file_1.docx]

**Appendix Table A1. Operational Definitions of the Researcher-Constructed CAMELS Proxies**

| **CAMELS Dimension** | **Operational Formula Used in This Study** | **Unit** | **Direction of Higher Values** | **Data Source** | **Interpretation** |
| --- | --- | --- | --- | --- | --- |
| C – Capital Adequacy | Total Equity / Total Assets | Ratio | Stronger bank soundness | Audited annual financial statements | Higher values indicate a stronger capital base and greater capacity to absorb losses. |
| A – Asset Quality | Allowance for Impairment of Receivables / Net Revenue | Ratio | Greater vulnerability | Audited annual financial statements | Higher values indicate greater impairment exposure and weaker asset quality. |
| M – Management Efficiency | Net Income / Total Revenue | Ratio | Stronger bank soundness | Audited annual financial statements | Higher values indicate greater efficiency in converting revenue into net income. |
| E – Earnings | Net Income / Total Assets (ROA) | Ratio | Stronger bank soundness | Audited annual financial statements | Higher values indicate stronger profitability and earnings performance. |
| L – Liquidity | Quick Ratio | Ratio | Stronger bank soundness | Audited annual financial statements | Higher values indicate a greater capacity to meet short-term obligations. |
| S – Sensitivity to Market Risk | Long-Term Deposits / Total Deposits | Ratio | Greater risk exposure | Audited annual financial statements | Higher values indicate greater exposure to the sensitivity-to-risk dimension operationalized in the study. |

Note. All CAMELS measures are researcher-constructed financial-ratio proxies derived from publicly available audited annual financial statements and are not official supervisory ratings. The operational formulas were adapted from Bawaneh and Dahiyat (2019).

**Appendix Table A2. Detailed Coding Guidance for the Eleven-Item Voluntary Disclosure Index**

| **Disclosure Category** | **Qualifying Disclosure Example** | **Non-Qualifying/Boilerplate Example** | **Principal Source Location** |
| --- | --- | --- | --- |
| Financial inclusion | Specific service, programme, branch initiative, target group, or measurable activity expanding access to finance | “The bank is committed to serving all members of society” | Annual report; sustainability/CSR section; strategic review |
| SME financing | Specific SME credit facility, programme, financing amount, policy, or partnership | “SMEs are important to the economy” | Annual report; lending portfolio; management discussion |
| CSR | Identifiable community programme, expenditure, partnership, or implemented activity | General claim that the bank is socially responsible | CSR/sustainability section |
| Customer satisfaction | Survey, complaint mechanism, response indicator, or customer-experience programme | “Customer satisfaction is our priority” | Customer-service or governance section |
| Forward-looking plans | Identifiable future strategy, target, expansion, investment, or implementation plan | “The bank looks forward to continued success” | Chairman/CEO statement; strategic review |
| Digital-platform usage | Named digital platform, service, user statistics, transactions, or implementation information | General claim of being digitally innovative | Digital-banking/operations section |
| Cybersecurity policy | Specific cybersecurity policy, control, committee, system, training, or risk procedure | “The bank takes security seriously” | Risk-management/governance section |
| Customer-data protection | Specific privacy, confidentiality, data-governance, or protection procedures | General commitment to confidentiality without identifiable procedure | Governance/risk/data-protection section |
| Gender diversity | Female representation data, policy, programme, target, or governance disclosure | Generic equality statement without identifiable information | Governance/human-resources section |
| Green financing | Green lending product, environmental policy, financing programme, amount, or eligibility criteria | General environmental commitment | Sustainability/product/credit section |
| Human-capital development and management continuity | Formal training programme, professional-development activity, succession plan, or explicit management-continuity disclosure | General claim that employees are valuable assets | Human-resources/governance section |
